# Clinical pharmacy services in mental health in Europe: a commentary paper of the European Society of Clinical Pharmacy Special Interest Group on Mental Health

**DOI:** 10.1007/s11096-023-01643-4

**Published:** 2023-09-27

**Authors:** Matej Stuhec, M. Hahn, I. Taskova, I. Bayraktar, I. Fitzgerald, L. Molitschnig, A. Tatarević, N. Lindner, L. Agnoletto, F. Alves da Costa

**Affiliations:** 1 Department of Pharmacology, Faculty of Medicine Maribor, University of Maribor, Maribor, Slovenia; 2 Present Address: Department of Clinical Pharmacy, Ormoz Psychiatric Hospital, Ormoz, Slovenia; 3 Department of Mental Health, Varisano Hospital Frankfurt Hoechst, Frankfurt, Germany; 4 Department of Psychiatry, Psychosomatics and Psychotherapy, University Hospital Frankfurt, Frankfurt, Germany; 5 Psychiatric Hospital Bohnice, Prague, Czech Republic; 6 Department of Applied Pharmacy, Faculty of Pharmacy, Masaryk University, Brno, Czech Republic; 7 Department of Clinical Pharmacy, Faculty of Pharmacy, Hacettepe University, Ankara, Turkey; 8 Pharmacy Department, St Patrick’s University Hospital, Dublin 8, Ireland; 9 School of Pharmacy, University College Cork, Cork, Ireland; 10 Pharmacy Department, Hospital of Elisabethians, Graz, Austria; 11 Pharmacy Bobanović Vujnović, Pula, Croatia; 12 Pharmacy Department, Vienna General Hospital-Medical University Campus, Vienna, Austria; 13 Hospital Pharmacy, Rovigo Hospital, Rovigo, Italy; 14 Research Institute for Medicines, Faculty of Pharmacy, University of Lisbon, Lisbon, Portugal

**Keywords:** Clinical pharmacist interventions, Clinical pharmacy in mental health, European society of clinical pharmacy, Mental illnesses and pharmacy, Pharmaceutical services in mental health, Polypharmacy and clinical pharmacy, Psychotropics and clinical pharmacy

## Abstract

A large proportion of the world’s disease burden is attributable to mental illnesses. Although effective interventions are available, many patients still have limited access to evidence-based treatments. Aside from access, treatment gaps, including inappropriate medication selection and monitoring, are also routinely recognised. Mental health clinical pharmacists can help address these gaps and enable patients to receive optimised pharmaceutical care, particularly appropriate medication selection and monitoring. The European Society of Clinical Pharmacy (ESCP) Special Interest Group on Mental Health was established to improve standardised service provision in mental health settings across Europe. The Special Interest Group identified significant barriers (predominantly associated with reimbursement and position within the multidisciplinary team) to effective pharmaceutical care amongst those with mental illnesses. This commentary presents recommendations to address these gaps through improved mental health clinical pharmacy service provision.

## Background

Mental illnesses represent one of the top ten causes of the overall global disease burden. The disease burden of mental illnesses (measured in disability-adjusted life-years (DALYs)) increased from 80.8 million in 1990 to 125.3 million in 2019, and the proportion of global DALYs attributed to mental illnesses increased from 3.1 to 4.9% [1]. Wittchen et al. reported that the most prevalent mental illnesses in 2010 in Europe were anxiety disorders (14.0%), insomnia (7.0%), major depression (6.9%), somatoform disorders (6.3%), and dementia (1–30%, depending on age). These authors estimated that 38.2% of the European population suffers from mental illnesses [2]. According to the world mental health report, in 2019, there were already 970 million people living with mental illnesses, with approximately 60% of those attributable to anxiety and depressive disorders [3]. The change in prevalence of these disorders increased by 26% in the first year of the COVID-19 pandemic [4]. Although effective pharmacological and non-pharmacological treatments are available, more than 75% of people in low- and middle-income countries receive no treatment [5]. In addition to access barriers, for those patients who receive treatment, it is often not considered optimal [4–6], representing a significant management gap. This gap often includes the absence of effective collaboration between different healthcare specialists (physicians, pharmacists, nurses, psychologists, etc.), as indicated by the most current National Institute for Health and Care Excellence (NICE) guidelines on depression treatment [7].

The European Society of Clinical Pharmacy (ESCP) Special Interest Group (SIG) on Mental Health is a members-only subgroup of ESCP. The SIG was established in 2022, and this commentary paper is one of the first outputs of the group. This group consists of established psychiatric clinicians with many years of experience in both clinical and research activities. The activities and aims of the group are focused on the optimised evidence-based treatment and prevention of a range of mental illnesses and are described in detail on the ESCP website. The SIG gives specific recommendations to all ESCP members and global readers to optimize the treatment and prevention of mental illnesses through various publications and appropriate forums [8].

This paper will also discuss the benefits of mental health clinical pharmacist specialists placed within the multidisciplinary psychiatric team on various clinical outcomes.

## Gaps in the pharmacological management of mental illnesses

Most recommendations and guidelines influencing pharmacotherapy practice within psychopharmacology are based primarily on the results of randomized controlled trials (RCTs) [9]. Within psychiatry, this can represent a significant issue in assessing the effectiveness of treatments in the typically diverse patient populations presenting in practice. Most patients with mental illnesses, particularly elderly patients, are not represented in typical RCTs, limiting evidence extrapolation [9]. An additional challenge in the care of these patients is that psychotropics are often prescribed within primary care settings from areas beyond psychiatry. This issue represents one barrier to patients accessing guideline-consistent care, which was seen in the Netherlands, where only 27% of patients with anxiety disorders received guideline-consistent care in primary care settings [10]. Similar findings were reported for depression treatment in the USA and Slovenia [6, 11].

In a German naturalistic study of outpatients diagnosed with depression (n = 153), most participants were not treated according to treatment guidelines. This included the absence of a treatment change after 4–8 weeks of non-response and the absence of therapeutic drug monitoring [12]. Higher adherence to guidelines was also associated with a higher remission rate in another German study, which suggests that treatment compliant with guidelines is to be favored over other treatment strategies [13]. Patients receiving treatment for mental illnesses in primary care also experienced high rates of drug–drug interactions, prescriptions of potentially inappropriate medications (PIMs) amongst elderly patients, and irrational polypharmacy, which can lead to poor clinical and economic outcomes [11, 14–18]. Stuhec and Zorjan found, in a multicentric study in Slovenia which included three primary care centers, that PIMs represented 9.5% of all medications taken by elderly patients with mental illnesses. PIM prevalence was very high (77.6% of patients were prescribed at least one PIM), and the most frequent PIMs were benzodiazepines and nonbenzodiazepine hypnotics, followed by antipsychotics [14].

In a Portuguese study conducted by Aguiar et al., which included elderly patients with a demonstrated burden of polypharmacy both in primary care and long-term facilities, nearly 60% of patients were identified as taking a PIM with a risk of cardiac and cerebrovascular adverse events (CCVAE). The pharmacotherapeutic group most frequently involved in PIMs identified in a subsample of long-term facility residents were antipsychotics, representing 38.7% of all PIMs with a risk of CCVAE [15]. Stuhec et al. found that, in primary care, psychotropics were the most frequent medication group involved in potentially severe drug–drug interactions, with more than 50% of patients affected. Psychotropics commonly implicated included escitalopram, clozapine, haloperidol, quetiapine and sulpride. In general population, psychotropics are most frequently linked to medication-related problems (PIMs and drug–drug interactions) [19]. Similar results have been observed in residential care settings, where benzodiazepines and antipsychotics are continuously used for non-approved indications despite important warnings highlighting associated risks and restricted use [20, 21]. Authors found that benzodiazepines and antipsychotics were used by 54% and 33% of all long-term care facility residents (n = 1730). They reported an extremely high prevalence of psychotropic prescriptions and frequent duplication of therapy [20]. Similar results were found in Austrian long-term care facilities (1844 residents, 48 nursing homes), where 50% of PIMs were associated with psychotropic medications and 50% of patients were treated with at least one psychotropic PIM. They found an extremely high prevalence (25.9% residents) of low-potency antipsychotic prothipendyl as well as benzodiazepine use for insomnia treatment and dementia management [21], despite warnings of higher mortality associated with antipsychotic use amongst elderly [22].

Aguiar et al. also explored the underlying mechanisms that lead to more serious adverse events associated with psychotropic medications by assessing reports on the World Health Organisation (WHO) Global Individual Case Safety Report (ICSR) database, VigiBase. They found that antipsychotics with high affinity for alpha-1 adrenergic receptors, histamine H1 receptors, muscarinic M1 receptors, and 5-HT2A receptors, and with a high risk of metabolic side effects may explain the occurrence of major adverse cardiovascular and cerebrovascular events (MACCE) [23].

Children and adolescents are another important patient group significantly affected by mental illnesses necessitating pharmacological management. There is also evidence of suboptimal pharmacological treatment of mental illnesses in this population. For example, Stuhec et al. found that many young patients with ADHD are not adequately treated (for example, a preference for the first line-use of atomoxetine over stimulant prescription was demonstrated) [24]. Collectively, these results highlight the urgent European-wide need for standardised and effective interventions aimed at optimising psychotropic use across patient groups and settings.

Hospital psychiatric settings coordinate the psychiatric care of those with acute and chronic illnesses, the latter including treatment-resistant cases. Pharmaceutical care for these patients can be complex. For example, in managing resistant cases of bipolar affective disorder, often a combination of a mood stabiliser, antipsychotic, and antidepressant is required to achieve symptom resolution and remission. In schizophrenia, antipsychotic treatment is the mainstay of the treatment. Effective antipsychotic treatment has been associated with several improved outcomes, including lower all-cause mortality and reduced risk of hospitalisation. Among those with treatment-resistant schizophrenia, clozapine is the most effective antipsychotic [25–27]. Although clozapine is recommended for first-line treatment amongst this cohort, its use in clinical practice remains suboptimal. This includes using clozapine later than indicated in many European countries, and often, antipsychotic polypharmacy is being prescribed before clozapine is considered, despite treatment guidelines recommending clozapine first [25, 28, 29].

In those whose illness is not considered treatment-resistant, long-acting injectable antipsychotics (LAI-antipsychotics) have been associated with improved treatment adherence and long-term outcomes, including reduced hospitalisations and mortality compared to oral preparations of the same antipsychotic [26, 27]. However, rates of LAI-antipsychotic prescriptions in Europe vary and remain far from optimal, given their unique benefits and associated recommended use in treatment guidelines [30]. Side effect monitoring associated with antipsychotic treatment, particularly metabolic side effects, remains consistently suboptimal despite the known prominent impact of metabolic side effects on antipsychotic adherence and future willingness to engage in treatment [31]. Non-adherence with recommended physical health monitoring amongst those taking antipsychotics is common, despite the contribution of cardiometabolic diseases to the mortality gap seen amongst those living with severe mental illnesses [26, 31]. For example, in one study in the UK, only 25% and 10% of patients initiating second-generation antipsychotics were screened for glucose and lipid abnormalities, respectively [31]. Additionally, non-adherence with treatment recommendations amongst those with schizophrenia includes antipsychotic treatment with multiple antipsychotics (antipsychotic polypharmacy = APP). For example, in one Slovenian psychiatric hospital, three antipsychotics were concomitantly prescribed to 22% of patients and two antipsychotics to 47% of patients [32, 33]. In psychiatric hospitals, different antipsychotic polypharmacy combinations, including combinations of low-dose antipsychotics, have been observed [34].

Several reasons may contribute to the range of suboptimal treatment practices. An international study conducted with healthcare professionals (physicians, pharmacists, and nurses) to assess knowledge and practices in managing medication complexities in elderly patients with mental illnesses showed that although most professionals felt confident, only a minority produced a good knowledge score in an assessment test. The study used clinical vignettes to evaluate performance, and those referring to dementia care obtained lower scores across all professional groups assessed, suggesting a more significant investment is needed in this area [35]. The main barriers to better medication management identified included process barriers, such as time, and structural barriers, including information systems deemed unfit for practice. In a subsequent study, Aguiar et al. identified through an e-Delphi process the most important patient-related features (PRF) that should be available in clinical records to support healthcare professionals in optimal medication management. They reported that even though many PRFs were rated as clinically relevant, some were identified as frequently missing from medical records [36]. There is positive evidence that a clinical pharmacy service for patients with mental illnesses has clinical and economic benefits and can address some of the previously mentioned gaps in practice [37–42]. These results highlight the urgent European-wide need for standardised and effective interventions to optimise psychotropic use across many patient groups and in varyious settings.

## Clinical pharmacy in the pharmacological management of mental illnesses

Clinical pharmacy services have been shown to reduce medication-related problems in patients with mental illnesses [37–42]. As demonstrated in studies conducted in the USA, clinical pharmacists as collaborative prescribers, or when conducting medication reviews, can lead to better clinical outcomes, including improved medication adherence [43–45]. Medication reviews provided by clinical pharmacists in primary care settings and long-term care facilities in patients with mental illnesses decreased the number of PIMs (20–50% decrease), the total number of medications (10% or more decrease) and potential drug–drug interactions (30–79% decrease). Medication reviews by clinical pharmacists also improved adherence to treatment guidelines and quality of life [11, 14, 46, 47]. In Scotland, researchers found that clinical pharmacists as independent prescribers in primary care improved clinical outcomes in the management of depression and anxiety disorders [48]. Clinical pharmacists, as a part of the multidisciplinary team in psychiatric hospitals, can also positively impact medication-related problems (e.g., reduce potential drug–drug interactions, reduce PIMs, reduce the total number of medications prescribed and aid in greater levels of identification of medication-related problems) [47, 49]. Stuhec and Tement found that clinical pharmacists, as multidisciplinary team members on psychiatric hospital wards, can recognize many medication-related problems, which means that together with psychiatrists, they can identify more problems than working independently. The acceptance of proposed pharmacist recommendations amongst psychiatrists was very high (94%), demonstrating recognition by psychiatrists of the relevance of pharmacists’ interventions [49]. In another study in the USA, researchers showed that clinical pharmacist interventions upon discharge could reduce APP use and increase the proportion of patients prescribed antipsychotic monotherapy in a psychiatric hospital (% of patients prescribed two or more antipsychotics at discharge declined from 33.9% at baseline (132 of 389 patients) to 21.8% after delivery of the educational modules (44 of 202 patients, *P* = 0.002) [50].

From the perspective of patients and other professionals caring for patients with mental illnesses, the clinical pharmacist is a legitimate and valuable member of the team [51]. A study published by Stuhec and Lah assessed clinical pharmacists' proposed recommendations to general practitioners on a medication review form. The authors reported that all accepted interventions except one (99.1%) were maintained 6 months after implementation and demonstrated the recognition of the value of clinical pharmacists' recommendations beyond the acute psychiatric setting. The multidisciplinary collaboration represents one of the most important approaches to reducing irrational polypharmacy in primary care and long-term facilities [11, 14]. The Eichberger Modell^**©**^ was developed in Germany in the University Psychiatric Clinic to assist with implementing psychiatric pharmacy services. In this model, the clinical pharmacist is part of the healthcare team and actively participates in pharmacotherapy selection and monitoring in a psychiatric hospital [52]. In the USA, mental health clinical pharmacists are well integrated into the psychiatric multidisciplinary team in some mental health departments. In a retrospective observational study including patients on clozapine, the pharmacist-led clinic had significantly higher metabolic and cardiovascular monitoring rates compared to the group without pharmacists. Service providers were also satisfied with the clinical pharmacist service [53]. Positive evidence of clinical pharmacists' interventions on outcomes for patients with mental illnesses has been summarized in a systematic review (n = 64 publications), where the authors found a positive impact on some outcomes (e.g., drug–drug interactions and medication adherence) [54].

Although there is positive evidence of the benefits of integrated clinical pharmacy services in the treatment of mental illnesses within both inpatient and outpatient settings, a mental health clinical pharmacy service is still not consistently provided in many European countries. Most countries do not have a well-established and appropriately reimbursed clinical pharmacy service that is provided on a standardised basis to patients with mental illnesses [49, 54]. Subsequently, practice varies substantially across Europe, affecting the quality of mental health care patients receive.

## Requirements for establishing mental health clinical pharmacy services across Europe

The paper published by Moura et al. shows varying legislation, clinical practices and reimbursement agreements in different European countries in the provision of mental health clinical pharmacy services. These problems have previously been highlighted, including the fact that each country defines the position, roles and need for clinical pharmacists working independently in their national legislation and, the healthcare system is thus structured accordingly [55]. Without adequate legislative and regulatory support, pharmacists are underutilized. Resolution CM/Res(2020)3, which includes a legal framework for 39 European member states for the promotion and implementation of the concept of pharmaceutical care and related services in health systems at a national level is a useful tool to support advocacy work [56].

Appropriate reimbursement models will also be required to support the standardised integration of clinical pharmacy services within mental health settings (e.g., psychiatric hospitals) and mental health teams. Successful support for reimbursement has been demonstrated through pilot assessments; for example, a psychiatric hospital in Germany (Eichberger Modell^**©**^) or in Slovenian pilot at national level [52, 57]. Germany was also successful in 2022 in achieving remuneration for five services in community pharmacies (not focused on mental health), demonstrating positive clinical outcomes in all patients [58]. It would be beneficial if other countries considered adopting a similar course of action.

Services in mental health care are not standardised, resulting in inequitable service provision across patients. ESCP could support countries in clinical pharmacy standardization and development [59, 60]. Clinical pharmacists are not uniformly part of multidisciplinary psychiatric teams, which reduces their effectiveness and opportunities for interventions to benefit patient care. This is especially important because clinical pharmacists in mental health institutions can recognize more medication-related problems than those working alone [49]. Pharmacist-led services lead to fewer medication errors in the transition of care [61]. Medication reconciliation on admission and discharge and seamless care are established in Slovenia and Portugal, but the national reimbursement is provided only in Slovenia. Countries could try to develop similar standardised advanced clinical pharmacy services at points of transition of care. Integration of clinical pharmacists in multidisciplinary teams within primary care is also an area requiring service development, as seen in the UK and Slovenia, and more recently in Portugal according to the Declaration of Astana, which aims to ensure that everyone in primary care can enjoy the highest possible standard of health [57, 62, 63]. Again, other European countries should look at these countries as models for developing and seeking reimbursement for these services.

Teaching and research within psychiatric pharmacy practice are additional important topics not covered in this paper. As an area of specialization, mental health clinical pharmacy should be developed and taught to a high standard, meaning that residents need teaching centers and well-developed clinical pharmacist positions. Most countries require initial or further work in this regard (e.g., subspeciality or additional certification). They should follow the example of other countries, for example, the USA, where they can receive board certification as a psychiatric pharmacist [64]. Pharmacists can prescribe psychotropic medications in the UK. Positive impacts on clinical outcomes of depression and anxiety were observed in primary care in a study provided in the UK (n = 75), where the clinical pharmacist independent prescriber was compared to the standard practice (without pharmacist independent prescriber) [48]. The UK have significantly advanced clinical pharmacy services in Europe such as the pharmacist prescriber, which may be seen as a model for other countries to follow [48, 54].

## Conclusion

Clinical pharmacy in mental health is not well developed in most countries, so clinical pharmacists do not fill the necessary pharmacotherapy gaps in patients with mental illnesses. The ESCP SIG on Mental Health can help develop and promote advanced clinical pharmacy mental health care services in European countries and broader. This is the first commentary paper of this group, and research studies, aside with education projects are planned to contribute to leading-edge clinical pharmacy in mental health.
